# Assessing SPI and SPEI for drought forecasting through the power law process: A case study in South Sulawesi, Indonesia

**DOI:** 10.1016/j.mex.2025.103235

**Published:** 2025-02-19

**Authors:** Nurtiti Sunusi, Nur Hikmah Auliana

**Affiliations:** Depatment of Statistics Faculty of Mathematics and Natural Sciences Hasanuddin University, Jl. Perintis Kemerdekaan KM 10 Tamalanrea, Makassar 90245, Indonesia

**Keywords:** Drought forecasting, Power law process, Nonhomogeneous poisson process, Standardized precipitation index (SPI), Standardized Precipitation Evapotranspiration Index (SPEI), Standardized Precipitation Index (SPI), Standardized Precipitation Evapotranspiration Index (SPEI), Power Law Process and Nonhomogeneous Poisson Process

## Abstract

This study presents a method for assessing drought events by integrating Standardized Precipitation Index (SPI) and Standardized Precipitation Evapotranspiration Index (SPEI) into the Power Law Process (PLP) model. The method begins with identifying drought events based on SPI and SPEI, followed by the Cramér–von Mises goodness-of-fit test to ensure the drought data meets PLP assumptions. Parameter estimation is performed using Maximum Likelihood Estimation (MLE) with a time-truncated approach, treating drought as a random process within a defined observation period. Model validation is conducted by comparing actual drought events with predictions from the cumulative PLP function, while event probabilities are determined using the Nonhomogeneous Poisson Process. Applied to 24 regencies/cities in South Sulawesi, the method showed that 14 regions fit the PLP based on SPI, and 13 regions based on SPEI. Predictions indicate that over the next 12 months, drought will occur for one month based on SPI and two months based on SPEI. This method contributes to the development of drought monitoring and early warning systems, supporting mitigation and adaptation strategies in South Sulawesi.

The main contributions of this study include:

• The development of a novel methodological framework by integrating SPI and SPEI into the PLP for drought analysis
• Practical applications in drought early warning systems and drought risk management in South Sulawesi

## Graphical abstract

**Figure:**
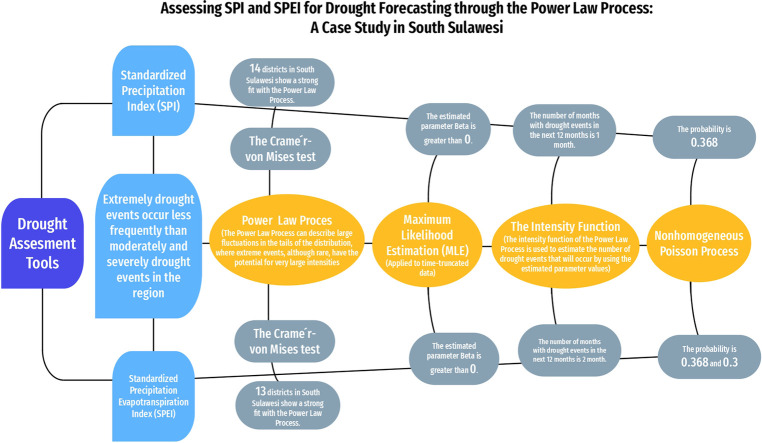

Specifications table

**Table:** 

Subject area:	Mathematics and Statistics
More specific subject area:	Statistical climate and Stochastic Process
Name of your method:	Standardized Precipitation Index (SPI), Standardized Precipitation Evapotranspiration Index (SPEI), Power Law Process and Nonhomogeneous Poisson Process
Name and reference of original method:	Auliana, N. H., Sunusi, N., and Herdiani, E. T., Analysis of meteorological drought periods based on the Standardized Precipitation Evapotranspiration Index (SPEI) using the Power Law Process approach, AIMS Environmental Science, 11(5) (2024), https://doi.org/10.3934/environsci.2024034
Resource availability:	The data used in this study are monthly rainfall data, maximum air temperature and minimum air temperature obtained based on single latitude and longitude coordinates for each region. The data can be accessed through the NASA POWER Project's Data Access Viewer on the following website: https://power.larc.nasa.gov/data-access-viewer/. The software used to analyze the data is DrinC, R Studio and ArcMap.

## Background

Precipitation deficiencies and temperature anomalies are the main factors that trigger various types of natural disasters that often cause huge losses to human life. Among these natural disasters, drought is the most common and complex phenomenon [1]. Drought has become a serious global challenge, affecting ecosystems, agriculture, water availability and human life. In Indonesia, South Sulawesi is one of the most vulnerable regions to the impacts of drought. As one of the nation's major food barns, the province relies heavily on stable climatic conditions to support water availability and agricultural activities. However, in recent years, the frequency and intensity of droughts in South Sulawesi have increased, threatening agricultural productivity and community welfare.

Drought can be viewed from various aspects, including: (1) Meteorological Drought, which is characterized by a lack of precipitation or rain from the average amount expected in a given period; (2) Hydrological Drought, which refers to the reduced flow and availability of water in water sources such as rivers, lakes, and reservoirs; (3) Agricultural Drought, which relates to the reduced soil moisture required for crop production; and (4) Socio-Economic Drought, which describes the impact of drought on the social and economic life of the community [2]. To monitor drought conditions, various drought indices can be used, including the Palmer Drought Severity Index (PDSI) [3], Deciles [4], Crop Moisture Index (CMI) [5], Surface Water Supply Index (SWSI) [6], Palmer Hydrological Drought Index (PHDI) [7], Standardized Precipitation Index (SPI) [8], and Standardized Precipitation Evapotranspiration Index (SPEI) [9].

Each drought index has characteristics that distinguish it from one another, both in terms of measurement methods, sensitivity to environmental variables, and the purpose of its use. Among these indices, SPI and SPEI are widely applied in Indonesia [[10], [11], [12], [13], [14], [15], [16]]. SPI uses rainfall data as the main parameter in its calculation [8]. Meanwhile, SPEI combines rainfall and potential evapotranspiration, making it more sensitive to climate change and the impact of increasing temperatures on drought [9]. The main advantage of both indices lies in their simplicity, as they only require rainfall and temperature data that are commonly available at many weather stations around the world. In addition, they offer flexibility in time scales, allowing the identification of droughts ranging from short-term to long-term. However, SPI and SPEI have limitations as they are only able to analyze drought conditions based on historical data, thus only providing information on droughts that have occurred or are occurring. Therefore, for more effective planning in anticipating future droughts, an appropriate method for predicting drought periods is needed.

In this study, we propose a new method that utilizes historical drought data based on SPI and SPEI in South Sulawesi. The data is integrated into the Power Law Process model. The choice of this model is based on the exploration of SPI and SPEI data, which shows that extreme drought events have a lower frequency compared to very dry and regular drought events. In statistics, this kind of pattern is known as a scale-frequency relationship, where high intensity events tend to occur less frequently than low to moderate intensity events. This pattern is in line with the characteristics described by the Power Law Process, which represents the scale-frequency relationship mathematically. The Power Law Process has the ability to describe large fluctuations in the tail of the distribution, where extreme events, although rare, have the potential for very large intensities [17].

To prove the assumption that drought occurrence patterns follow a Power Law distribution, we conducted a distribution fit test using the Cramér-von Mises test. This test aims to assess the extent to which the empirical data fits the assumed distribution, so that the validity of using the Power Law Process in modeling the probability of drought occurrence can be statistically verified. In addition, to perform parameter estimation, we used the Maximum Likelihood Estimation (MLE) method with a time-truncated approach. This approach was chosen because the drought data collection process based on SPI and SPEI is carried out within a predetermined time span. Thus, the data is assumed to be time-truncated data, where the number of drought events obtained in the observation period is a random variable. This approach is similar to the method used in system failure studies [18,19].

The novelty of this research lies in the integration of SPI and SPEI with the PLP model to predict drought periods, an approach that has never been applied before in the South Sulawesi region. The results of this study will provide an understanding of the differences in drought period characteristics based on SPI, which represents rainfall deficits, and SPEI, which considers the balance between rainfall and evaporation-transpiration. By adopting this method, the research results are expected to provide deeper insights into drought patterns in the region as well as offer more accurate prediction tools for water resource planners and policy makers to manage drought impacts more effectively.

## Method details

**Fig. 1:**
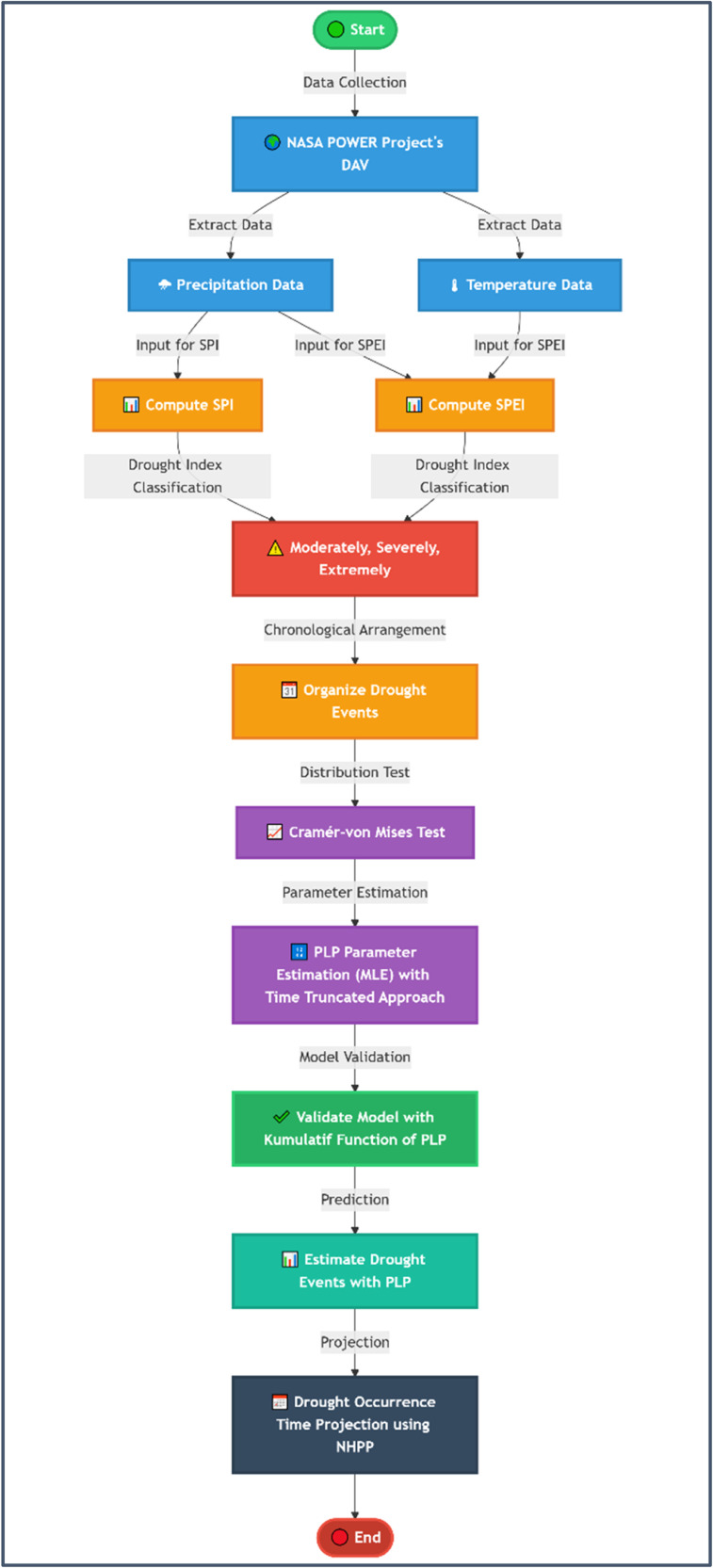
Flowchart methodology.

To ensure clarity and repeatability of the analysis, the methodological workflow was organized in a stepwise approach and supported by a flow chart detailed in Fig. 1. The analysis process began with the collection of rainfall and air temperature data, which were then used to calculate SPI and SPEI values. SPI and SPEI calculations were performed using a 1-month time scale, in accordance with the research needs to assess short-term drought. Next, SPI and SPEI values classified as moderately drought, severely drought, extremely drought were collected, and the times of occurrence were sorted chronologically within the observation time span. After that, the model was tested and the Power Law Process parameters were estimated using Maximum Likelihood Estimation (MLE). The results of this parameter estimation were used in the cumulative Power Law Process function to estimate the number of drought events during the observation period. After ensuring that the estimation results match the actual data in the period, the number of drought events in the future is estimated. To determine the probability of future drought events, a Nonhomogeneous Poisson Process is used based on the initial estimation of the projected number of drought events

### Observation areas and data collection

**Fig. 2:**
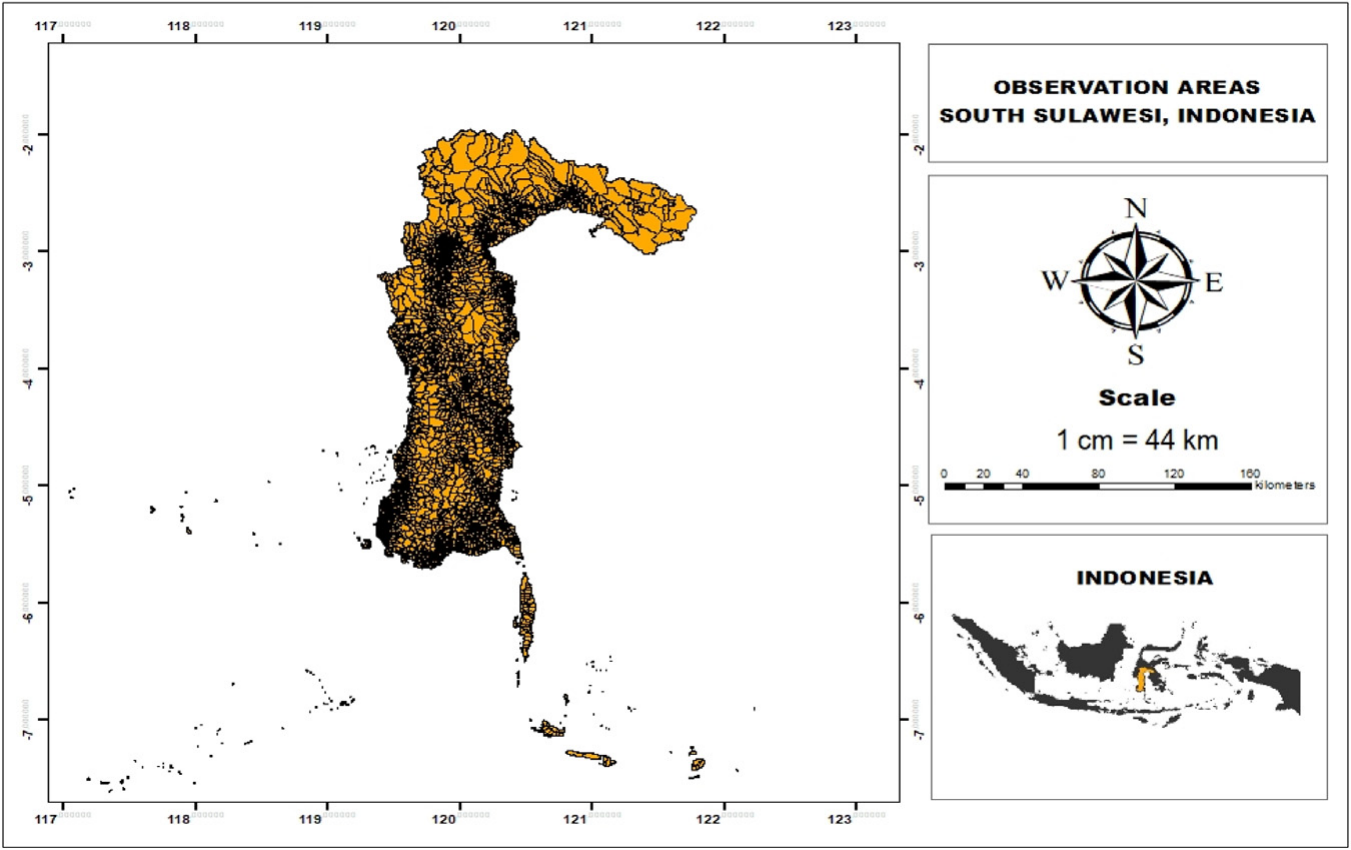
Observation Areas.

The study covered 24 districts/cities in South Sulawesi, which lies between latitude 0° 12′- 8′ N and longitude 116° 48′−122° 36′ E. The region borders West Sulawesi to the north, the Gulf of Bone and Southeast Sulawesi to the east, the Makassar Strait to the west, and the Flores Sea to the south (Fig. 2). South Sulawesi has a wet tropical climate with two main seasons, the rainy season and the dry season, which affect rainfall and temperature patterns. The meteorological data used includes monthly rainfall amounts, maximum air temperature, and minimum air temperature from January 1981 to December 2023.

Data for the period January 1981 through December 2022 were obtained from the NASA POWER Project (https://power.larc.nasa.gov/data-access-viewer/) in monthly format with a spatial resolution of 0.5° x 0.625°. However, monthly data for 2023 was not available from NASA POWER at the time of this study. However, daily data for 2023 was accessible, so monthly rainfall amounts were calculated manually by summing daily rainfall data. Similarly, monthly maximum and minimum air temperatures were determined manually by identifying the highest and lowest daily temperatures from the same daily dataset. This approach ensured the completeness and continuity of the dataset for the entire study period. The data collected forms the basis for calculating the Standardized Precipitation Index (SPI) and Standardized Precipitation Evapotranspiration Index (SPEI), which are used to analyze drought occurrence. In addition, the variety of data collection locations for each district/city in South Sulawesi, based on their respective latitude and longitude coordinates, ensured that the analysis covered the entire study area in a representative manner.

### Standardized precipitation index (SPI)

The Standardized Precipitation Index (SPI) is one of the most commonly used methods for measuring drought based on rainfall data. The SPI quantifies rainfall deficits or surpluses by transforming the rainfall distribution into a normal distribution, thus allowing comparison of drought conditions across different regions and time periods. In this study, the SPI was calculated using a 1-month time scale to assess short-term drought, which is particularly relevant for agricultural planning and water resource management in South Sulawesi.

The SPI calculation begins by converting the observed rainfall data to a gamma distribution using the following equation: [8]

(1) $$g\left(x\right)=\frac{1}{\beta^{\alpha }\Gamma \left(\alpha \right)}\;x^{\alpha -1}e^{-\frac{x}{\beta }}\;\text{for}\;x,\;\beta ,\alpha >0$$

The gamma function is obtained from:

(2) $$\Gamma \left(\alpha \right)=\int_{0}^{\infty }y^{\alpha -1}e^{-y}\;dy$$

to determine the parameters αand β, the following formula is used:

(3) $$\alpha =\frac{{\left(\bar{x\;}\right)}^{2}}{Sd^{2}}\;\text{and}\;\beta =\bar{\frac{x}{\alpha }}$$

With:

(4) $$\bar{x\;}=\frac{\sum x}{n}\;\text{and}\;Sd=\sqrt{\frac{{\left(x-\bar{x\;}\right)}^{\;2}}{n-1}}$$

The gamma distribution is undefined for x=0, therefore, for zero rainfall, the following cumulative probability is used:

(5) H(x)=q+(1−q)g(x)

q is the probability of zero rainfall, or q is the number of zero rainfall events divided by the amount of data. The calculation of the SPI value then converts the gamma distribution of rainfall into a normal distribution with the following equation:

For 0<H(x)≤0,5

(6) $$SPI=\;-\left(t-\frac{c_{0}+c_{1}t+c_{2}t^{2}}{1+d_{1}t+d_{2}t^{2}+d_{3}t^{3}}\right),$$

Where:

(7) $$t=\sqrt{ln\left(\frac{1}{{\left(H\left(x\right)\right)}^{2}}\right)}$$

For 0,5<H(x)≤1

(8) $$SPI=\;\left(t-\frac{c_{0}+c_{1}t+c_{2}t^{2}}{1+d_{1}t+d_{2}t^{2}+d_{3}t^{3}}\right),$$

Where:

(9) $$t=\sqrt{ln\left(\frac{1}{{\left(1-H\left(x\right)\right)}^{2}}\right)}$$

With:

$$c_{0}=2.515517\;d_{1}=1.432788$$

$$c_{1}=0.802853\;d_{2}=0.189269$$

$$c_{2}=0.010328\;d_{3}=0.001308$$

### Standardized precipitation evapotranspiration index (SPEI)

The Standardized Evapotranspiration Precipitation Index (SPEI) is a method developed to measure drought by considering not only rainfall but also evapotranspiration potential (ETP). Unlike SPI which is only based on rainfall, SPEI incorporates the influence of temperature, making it more sensitive to climate change and its impact on water availability. In this study, the SPEI was calculated using a 1-month timescale to assess short-term drought, which is particularly relevant for understanding the impacts of drought on agriculture and water resources in South Sulawesi. By considering the balance between rainfall and evapotranspiration, the SPEI provides a more comprehensive picture of drought conditions. The SPEI is standardized based on the log-logistic distribution probability density function with Eq: [9]

(10) $$f\left(D\right)=\frac{\beta }{\alpha }{\left(\frac{D-\gamma }{\alpha }\right)}^{\beta -1}{\left[1+\;{\left(\frac{D-\gamma }{\alpha }\right)}^{\beta }\right]}^{-2},\;\gamma >D>\infty$$

The parameters α, β, and γ in the log-logistic distribution are calculated using the l-moment method, with the Pearson III distribution parameters calculated as follows:

(11) $$\beta =\frac{2W_{1}-W_{0}}{6W_{1}-W_{0}-6W_{2}}$$

(12) $$a=\frac{\left(W_{0}-2W_{1}\right)\beta }{\Gamma \left(1+\frac{1}{\beta }\right)\Gamma \left(1-\frac{1}{\beta }\right)}$$

(13) $$\gamma =W_{0}-\alpha \Gamma \left(\frac{1+1}{\beta }\right)\Gamma \left(\frac{1-1}{\beta }\right)$$

Γ(β) is the gamma function distribution of β and W is the Probability Weighted Moments (PWMs) obtained from: [20]

(14) $$W_{s}=\frac{1}{N}\sum_{i=1}^{N}{\left(1-F_{i}\right)}^{s}\;D_{i}$$

with s as the number of PWMs, and is the estimated frequency calculated as:

(15) $$F_{i}=\frac{i-0,35}{N}$$

i is the range of observations sorted in ascending order, and N is the number of data used. $D_{i}$ is the climate water balance or deficit between rainfall and PET calculated as:

(16) $$D_{i}=CH_{i}-\text{PE}T_{i}$$

PET can be calculated using the Hargreaves method by considering the monthly average air temperature (T) as follows: [21]

(17) $$PET=\;0.0023\;Ra\;(\sqrt{T_{Max}-T_{Min})}\;\left(T_{Max}+17.8\right)$$

$T_{Max}$ is the maximum air temperature, $T_{Min}$ is the minimum air temperature and Ra is the solar radiation. The value of Ra is usually predicted from the latitude of the place and time of measurement. Alternative PWM estimators can be obtained by other methods, such as the unbiased estimator [22]:

(18) $$W_{s}=\frac{1}{N}\sum_{i=1}^{N}\frac{\left(\begin{array}{c} N-i\\ s \end{array}\right)Di}{\left(\begin{array}{c} N-i\\ s \end{array}\right)}$$

The probability function of the distribution of D in various time scales is calculated using the equation:

(19) $$F\left(D\right)={\left[1+\;{\left(\frac{\alpha }{D-\gamma }\right)}^{\beta }\right]}^{-1}$$

Based on this probability function, the SPEI can be calculated with the equation:

(20) $$SPEI=\;\left(t-\frac{c_{0}+c_{1}W+c_{2}W^{2}}{1+d_{1}W+d_{2}W^{2}+d_{3}W^{3}}\right)$$

With:

(21) $$W=\sqrt{-2\ln\left(P\right)\;}\;\text{for}\;P\;\leq \;0.5$$

(22) $$W=\sqrt{-2\ln\left(1-P\right)\;}\;\text{for}\;P\;\geq \;0.5$$

P is the probability of exceeding the D value calculated with:

(23) P=1−F(x)

The drought severity classification for SPEI is similar to SPI, as shown in Table 1.

**Table 1:** Drought classification based on SPI.

SPI Values	Classification
(- 0.99) – 0.99	Normal
(−0.1) – (−1.49)	Moderately Drought
(−1.5) – (−1.99)	Severely Drought
≤ - 2.00	Extremely Drought

### Nonhomogeneous poisson process

The Nonhomogeneous Poisson Process is a stochastic process model used to describe random events that occur at certain time intervals, with the intensity of the event changing over time. The Nonhomogeneous Poisson Process is a generalization of the Homogeneous Poisson Process and is often referred to as the Nonstationary Poisson Process. This is because the rate of occurrence in the Nonhomogeneous Poisson Process is not constant. In a Nonhomogeneous Poisson Process, the intensity or rate of occurrence is represented by an intensity function λ(t). The intensity function λ(t) describes how the rate of an event varies over time. A counting process {N(t),t≥0} is said to be a Nonhomogeneous Poisson Process with intensity function λ(t), t≥0, if: ([23]

a. N(0)=0,
b. {N(t),t≥0} has an independent increment*,*
c. P{N(t+h)−N(t)=1}=λ(t)+o(h), and
d. P{N(t+h)−N(t)≥2}=o(h), where h > 0 and o(h) is a small number that fulfils the condition$\;\lim_{h\to 0}\frac{o(h)}{h}=0.$

Nonhomogeneous Poisson process has a parameter λ(t)called intensity function defined as:

(24) $$m\left(t\right)=\int_{0}^{t}\;\lambda \left(t\right)\;dt$$

m(t) is the mean value function of the Nonhomogeneous Poisson Process. For t≥0 and n≥0, it is obtained:

(25) $$P\left(N_{\left(t\right)}=n\right)=\frac{\left[\int_{0}^{t}\lambda \left(t\right)dt\right]}{n!}\;exp^{-\int_{0}^{t}\lambda \left(t\right)dt}\;n=0,1,2\ldots n$$

### Power law process (PLP)

Power Law Process (PLP) is a specialized model in the category of Nonhomogeneous Poisson Process, where the intensity function is time dependent. PLP is widely used in system failure analysis. Other phenomena that also illustrate the Power Law model, such as extreme natural events including floods [24] and earthquakes [25,26], show that the model has wide applications in modelling infrequent but significant events. In this study, PLP is used to model drought events based on historical data, with the intensity function defined as: [18]

(26) $$\lambda \left(t\right)=\left(\frac{\beta }{\gamma }\right)\left(\frac{t}{\gamma }\right)\begin{array}{c} \beta -1\\ \; \end{array},\;\gamma >0,\;\beta >1,\;t>0$$

Meanwhile, the expected value based on Eq. (24) is given by the cumulative function of the Power Law Process as follows:

(27) $$m\left(t\right)=\left(\frac{t}{\gamma }\right)\begin{array}{c} \beta \\ \; \end{array},\;\gamma >0,\;\beta >1,\;t>0$$

To test the suitability of the Power Law model, the Cram$\overset{\prime }{e}$r-von Mises test is used with the hypothesis:

$H_{0}:$ Event intensity fits the *Power Law Process* model

$H_{1}:\;$Event intensity does not fit the *Power Law Process* model

The Cram$\overset{\prime }{e}$r-von Mises test statistic is expressed based on the following equation: [18]

(28) $$C_{R}^{2}=\frac{1}{12\left(n\right)}+\;\sum_{i=1}^{n}{\left(\bar{R}-\frac{2i-1}{2n}\right)}^{2}$$

$\bar{R}$ is the ratio power transformation given by:

(29) $$\bar{R}={\left(\frac{t_{i}}{t_{n}}\right)}^{\bar{\beta }}$$

$\bar{\beta }$ is an unbiased estimator given by:

(30) $$\bar{\beta }=\frac{\left(n-2\right)}{\sum_{i=1}^{n}\ln\left(\frac{t_{n}}{t_{i}}\right)}$$

The decision is $H_{0}$ accepted if the test statistic $C_{R}^{2}$ value based on the calculation is smaller than the critical value for the Cram$\overset{\prime }{e}$r-von Mises Test, which means that the *Power Law Process Model* is suitable. If the value of the test statistic $C_{R}^{2}$ is greater than the critical value for the Cram$\overset{\prime }{e}$r-von Mises Test then it is $H_{0}$ rejected, which means the model does not fit.

## The likelihood function

In Power Law Process analysis, there are two main types of data used, namely: [19].

1. Failure-truncated data: This data is obtained by specifying a certain number of events, where observations take place until a certain number of failures or events occur.
2. Time-truncated data: This data is obtained by setting a specific time period, where the number of events occurring within that period becomes a random variable.

In this study, the time-truncated approach was chosen because the drought data collection process based on SPI and SPEI is carried out within a predetermined time span. Thus, the data used reflects the number of drought events that occur within a certain observation period, not based on a certain number of events that must be achieved. To perform parameter estimation, we use the Maximum Likelihood Estimation (MLE) method which optimally estimates parameters based on observed data [18,27]. Suppose $t_{1},t_{2},t_{3},\ldots ,t_{n}$ is an independent random sample from a distribution with joint likelihood function $f(t_{1},t_{2},t_{3},\ldots ,t_{n};\beta ,\gamma )$ with n denoting the number of events occurring up to time $t_{i}$ for $0<t_{1}<t_{2}<t_{3}<\ldots <t_{n}$. If the joint likelihood function is expressed as a function of β,γ then the likelihood function is denoted as $L(t_{1},t_{2},t_{3},\ldots ,t_{n};\beta ,\gamma ).$ The likelihood function for the parameter β,γ is given as follows: [22]

(31) $$L\left(t_{1},t_{2},t_{3},\ldots ,t_{n};\beta ,\gamma \right)=\left(\prod_{i=1}^{n}\lambda \left(t_{i};\beta ,\gamma \right)\right)exp\left(-\int_{0}^{t_{n}}\lambda \left(t_{i};\beta ,\gamma \right)dt\right)$$

Based on Eq. (31), the likelihood function with intensity $\lambda (t_{i};\beta ,\gamma )$function is:

(32) $$L\left(t_{i};\beta ,\gamma \right)=\left(\prod_{i=1}^{n}\left(\frac{\beta }{\gamma }\right){\left(\frac{t_{i}}{\gamma }\right)}^{\begin{array}{c} \beta -1\\ \; \end{array}}\right)\;exp\left(-\int_{0}^{t_{n}}\left(\frac{\beta }{\gamma }\right){\left(\frac{t}{\gamma }\right)}^{\begin{array}{c} \beta -1\\ \; \end{array}}dt\right)$$

Based on Eq. (32), the logarithmic likelihood function of L(t;β,γ)=ln⁡(L(t;β,γ)) is:

(33) $$L\left(t;\beta ,\gamma \right)=n\ln\left(\beta \right)-n\beta \ln\left(\gamma \right)+\left(\beta -1\right)\sum_{i=1}^{n}\ln\left(t_{i}\right)-{\left(\frac{t_{n}}{\gamma }\right)}^{\beta }$$

Furthermore, Eq. (33) is derived from β and γ so that the maximum likelihood estimator is obtained as follows:

(34) $$\hat{\beta }=\frac{n}{\sum_{i=1}^{n}\ln\left(\frac{t_{n}}{t_{i}}\right)}$$

(35) $$\hat{\gamma }=\frac{t_{n}}{n^{\frac{1}{\hat{\beta }}}}$$

## Method validation

### Analysis of drought intensity distribution based on SPI and SPEI

As part of the method validation, we analyzed the characteristics of drought intensity obtained based on SPI and SPEI values in South Sulawesi. For this purpose, we used the classification system of McKee et al. (1993) [8], which divides drought intensity into three categories: Moderately Drought, Severely Drought and Extremely Drought. In this section, we aim to validate the finding that extreme dry events are less common than very dry and dry events.

**Fig. 3:**
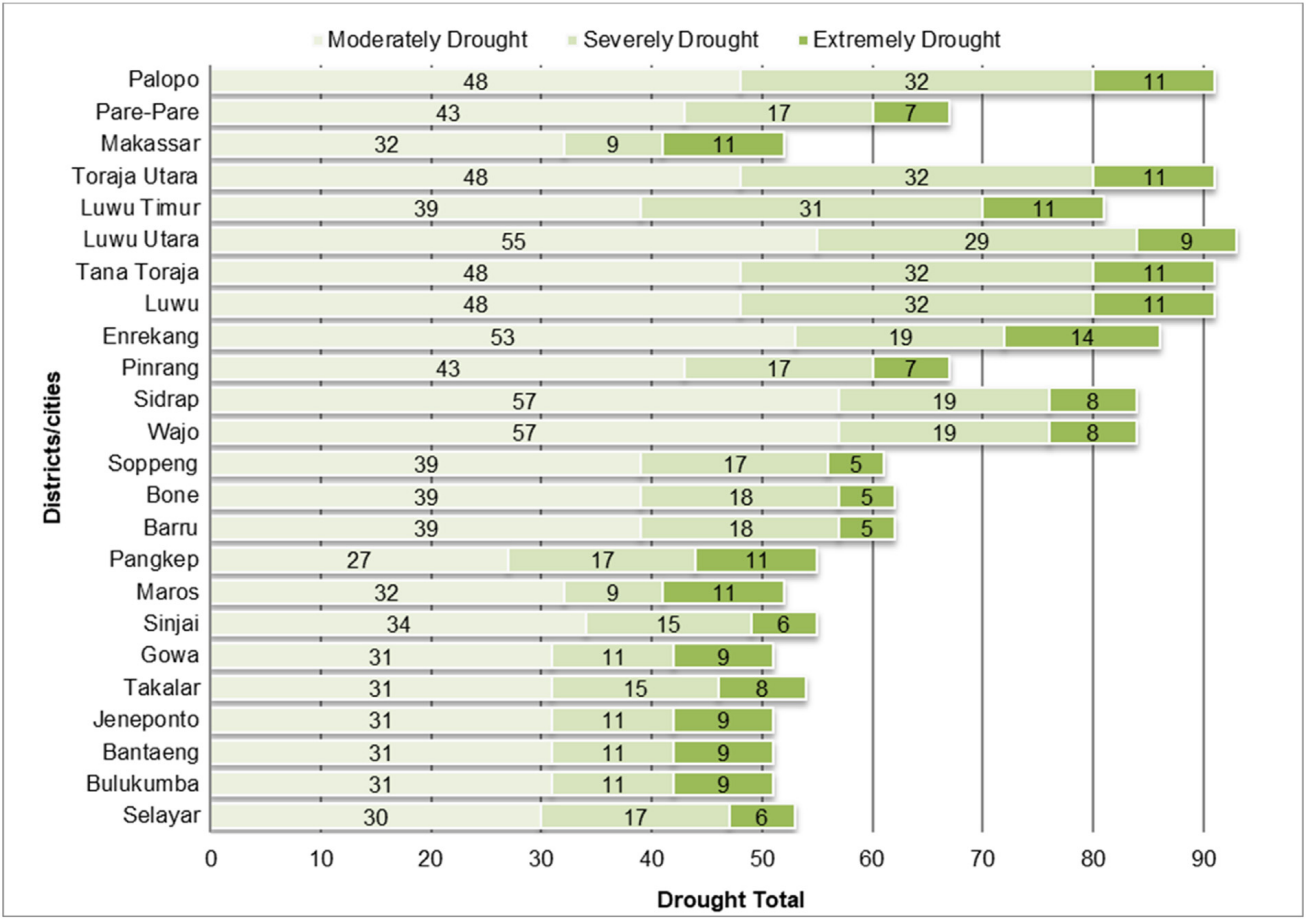
Distribution of SPI drought levels that have occurred in south sulawesi during observations 1981–2023.

**Fig. 4:**
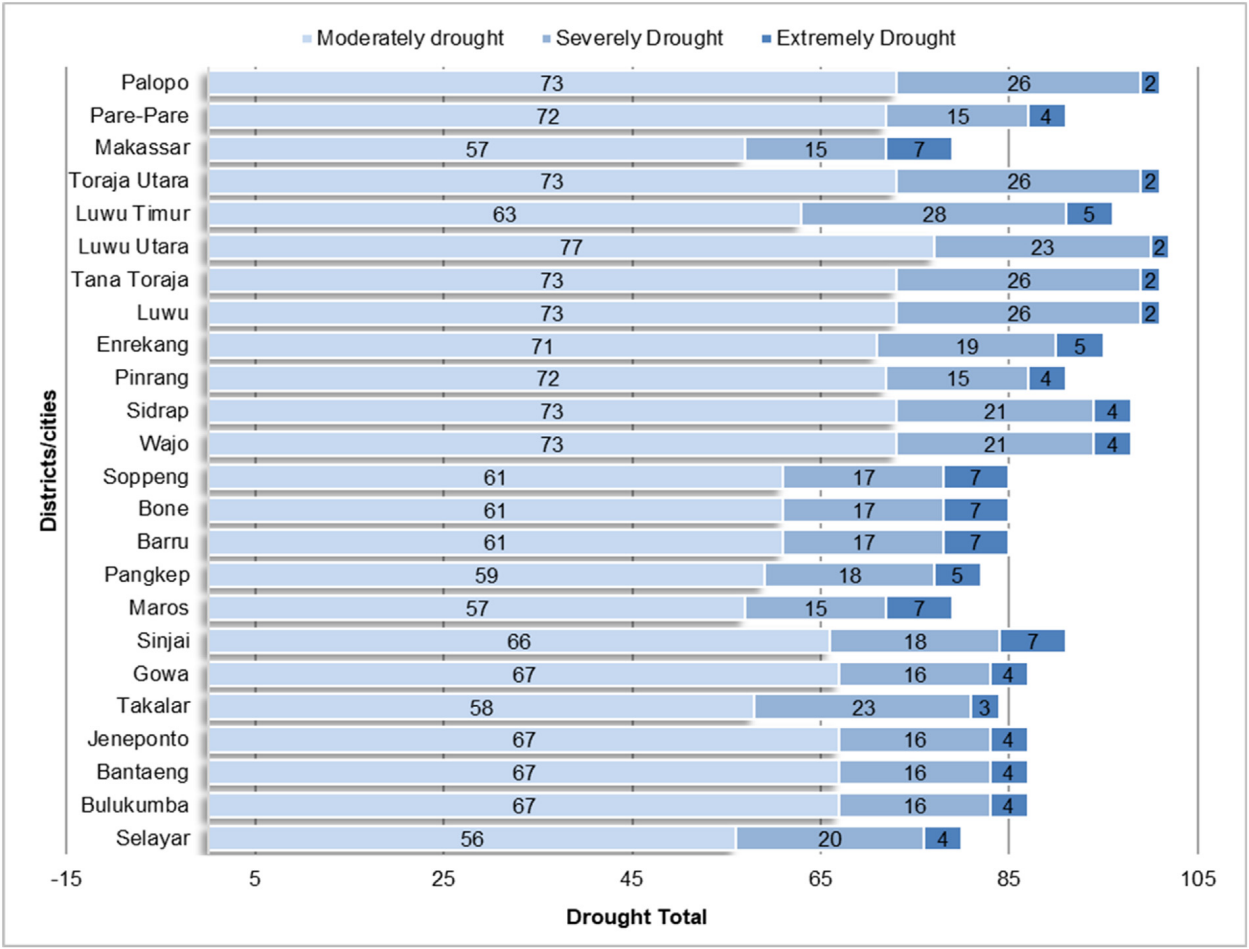
Distribution of SPEI drought levels that have occurred in south sulawesi during observations 1981–2023.

The classification results show that drought events with Moderately Drought and Severely Drought event intensities are more frequent than drought events that fall into the Extreme Drought category. This can be seen in Fig. 3, Fig. 4, which illustrate the number of drought events based on SPI and SPEI levels in each district/city in South Sulawesi. The data shows that Moderately Drought and Severely Drought events have a higher frequency in most areas, while Extremely Drought events are recorded less frequently, which is in line with the general characteristics of droughts that do not often reach extreme levels.

This analysis confirms that the SPI and SPEI classification methods are effective in describing the distribution of drought intensity, with Extremely Drought events occurring less frequently than regular droughts, supporting the validity of using these methods in the observed areas.

### Fit testing of the power law process model

The process of identifying drought intensity in South Sulawesi using SPI and SPEI provides insight into the pattern of drought, where Extremely Drought events occur less frequently than droughts in the ordinary Moderately Drought and Severely Drought categories. Based on these characteristics, the phenomenon of drought intensity can be interpreted within the framework of the Power Law Process model. To validate the fit of this model to the identified drought intensity data, fit testing was conducted using the Cramér-von Mises statistical test $\left(C_{R}^{2}\right)$.

In this test, the null hypothesis (*H*_0_) states that the drought intensity data follows the Power Law Process distribution, while the alternative hypothesis (*H*_1_) states that there is a mismatch between the data and the model. The Cramér-von Mises test is used to measure the level of fit between the data and the model by comparing the value of the test statistic $\left(C_{R}^{2}\right)$ against a predetermined critical value.

If the value of the $C_{R}^{2}$ test statistic is smaller than the critical value, the null hypothesis (*H*_0_) is accepted, indicating that the drought intensity data fits the Power Law model. Conversely, if $C_{R}^{2}$ is greater than the critical value, the alternative hypothesis (*H*_1_) is accepted, indicating that the Power Law model does not fit the observed drought intensity data.

The results of the Cramér-von Mises test are presented in Table 2, Table 3, which show the calculations from Eqs. (28) to (30) to assess the extent to which the model fits the data.

**Table 2:** Cramér-von Mises test results SPI.

**Cities/Regencies**	$C_{R}^{2}$	**Critical** **Value**	**Decision**
Selayar	0,12	0,22	$H_{0}$ Accepted
Bulukumba	0,05	0,22	$H_{0}$ Accepted
Bantaeng	0,05	0,22	$H_{0}$ Accepted
Jeneponto	0,05	0,22	$H_{0}$ Accepted
Takalar	0,07	0,22	$H_{0}$ Accepted
Gowa	0,05	0,22	$H_{0}$ Accepted
Sinjai	0,06	0,22	$H_{0}$ Accepted
Maros	0,21	0,22	$H_{0}$ Accepted
Pangkep	0,38	0,22	$H_{1}$ Accepted
Barru	0,12	0,22	$H_{0}$ Accepted
Bone	0,12	0,22	$H_{0}$ Accepted
Soppeng	0,15	0,22	$H_{0}$ Accepted
Wajo	0,55	0,22	$H_{1}$ Accepted
Sidrap	0,55	0,22	$H_{1}$ Accepted
Pinrang	0,11	0,22	$H_{0}$ Accepted
Enrekang	0,80	0,22	$H_{1}$ Accepted
Luwu	0,92	0,22	$H_{1}$ Accepted
Tana Toraja	0,92	0,22	$H_{1}$ Accepted
North Luwu	1,13	0,22	$H_{1}$ Accepted
East Luwu	0,63	0,22	$H_{1}$ Accepted
North Toraja	0,92	0,22	$H_{1}$ Accepted
Makassar	0,21	0,22	$H_{0}$ Accepted
Pare-Pare	0,11	0,22	$H_{0}$ Accepted
Palopo	0,92	0,22	$H_{1}$ Accepted

**Table 3:** Cramér-von Mises test results SPEI.

**Cities/Regencies**	$C_{R}^{2}$	**Critical** **Value**	**Decision**
Selayar	0,10	0,22	$H_{0}\;$Accepted
Bulukumba	0,05	0,22	$H_{0}\;$Accepted
Bantaeng	0,05	0,22	$H_{0}\;$Accepted
Jeneponto	0,05	0,22	$H_{0}\;$Accepted
Takalar	0,13	0,22	$H_{0}\;$Accepted
Gowa	0,05	0,22	$H_{0}\;$Accepted
Sinjai	0,05	0,22	$H_{0}\;$Accepted
Maros	0,12	0,22	$H_{0}\;$Accepted
Pangkep	0,22	0,22	$H_{0}\;$Accepted
Barru	0,15	0,22	$H_{0}\;$Accepted
Bone	0,15	0,22	$H_{0}\;$Accepted
Soppeng	0,15	0,22	$H_{0}\;$Accepted
Wajo	0,35	0,22	$H_{1}\;$Accepted
Sidrap	0,35	0,22	$H_{1}$ Accepted
Pinrang	0,23	0,22	$H_{1}$ Accepted
Enrekang	0,80	0,22	$H_{1}$ Accepted
Luwu	1,07	0,22	$H_{1}$ Accepted
Tana Toraja	1,07	0,22	$H_{1}$ Accepted
North Luwu	1,13	0,22	$H_{1}$ Accepted
East Luwu	0,92	0,22	$H_{1}$ Accepted
North Toraja	1,07	0,22	$H_{1}$ Accepted
Makassar	0,12	0,22	$H_{0}$ Accepted
Pare-Pare	0,23	0,22	$H_{1}$ Accepted
Palopo	1,07	0,22	$H_{1}$ Accepted

The results of the drought intensity fit test based on SPI show that 14 districts/cities in South Sulawesi fit the Power Law Process model, while based on SPEI there are 13 districts/cities that fit. However, some areas do not fit this model, although exploratively the pattern of drought occurrence shows that extreme events are less frequent than dry or very dry categories (see Fig. 3, Fig. 4). This mismatch suggests that the pattern of drought intensity in these areas does not follow the distribution expected in the PLP model, which may indicate that other factors are influencing the drought dynamics in these areas. Therefore, further analysis is required to determine a more appropriate model so that mitigation strategies can be adjusted accordingly. In this study, we will focus on regions that fit the PLP model to estimate the number of drought months in the future and support more accurate mitigation planning.

### Parameter estimation and validation process of power law model

Parameter estimation of the Power Law Process model is performed using the Maximum Likelihood Estimation (MLE) method, which is calculated based on Eqs. (34) and (35). In this context, MLE is used to find the best parameters that maximize the likelihood of the observed data following a Power Law distribution. The data used consists of chronologically ordered drought event times, as well as data on the number of identified drought events (n) and observation time intervals (t) in each region.

This estimation process aims to measure the fit of the model with the data, by optimizing the model parameters so that the Power Law distribution can describe the phenomenon of drought events more accurately. The estimated parameters are then used to validate whether the Power Law Process model is an appropriate representation of the observed drought occurrence pattern in the data.

The parameter estimation results obtained through MLE are presented in Table 4, Table 5, which show the estimated parameter values for each region as well as the extent to which the Power Law model can describe the distribution of drought events in the region. The validity of the model can be further evaluated by comparing these estimation results with model fit tests such as the Cramer-von Mises test or other methods used to test whether the model provides a valid representation of the data.

**Table 4:** Parameter estimation value of power law process for SPI.

**Cities/Regencies**	$\hat{\beta }$	$\hat{\gamma }$
Selayar	0,92	6,96
Bulukumba	0,88	6,03
Bantaeng	0,88	6,03
Jeneponto	0,88	6,03
Takalar	1,09	13,22
Gowa	0,88	6,03
Sinjai	0,97	8,17
Maros	0,85	4,93
Barru	0,87	4,61
Bone	0,87	4,61
Soppeng	0,86	4,35
Pinrang	0,76	2,07
Makassar	0,85	4,93
Pare-Pare	0,76	2,07

**Table 5:** Parameter estimation value of power law process for SPEI.

**Cities/Regencies**	$\hat{\beta }$	$\hat{\gamma }$
Selayar	1,03	7,28
Bulukumba	0,92	4,06
Bantaeng	0,92	4,06
Jeneponto	0,92	4,06
Takalar	1,07	8,32
Gowa	0,92	4,06
Sinjai	1,01	5,92
Maros	0,88	3,65
Pangkep	0,79	1,98
Barru	0,88	3,25
Bone	0,88	3,25
Soppeng	0,88	3,25
Makassar	0,88	3,65

**Fig. 5:**
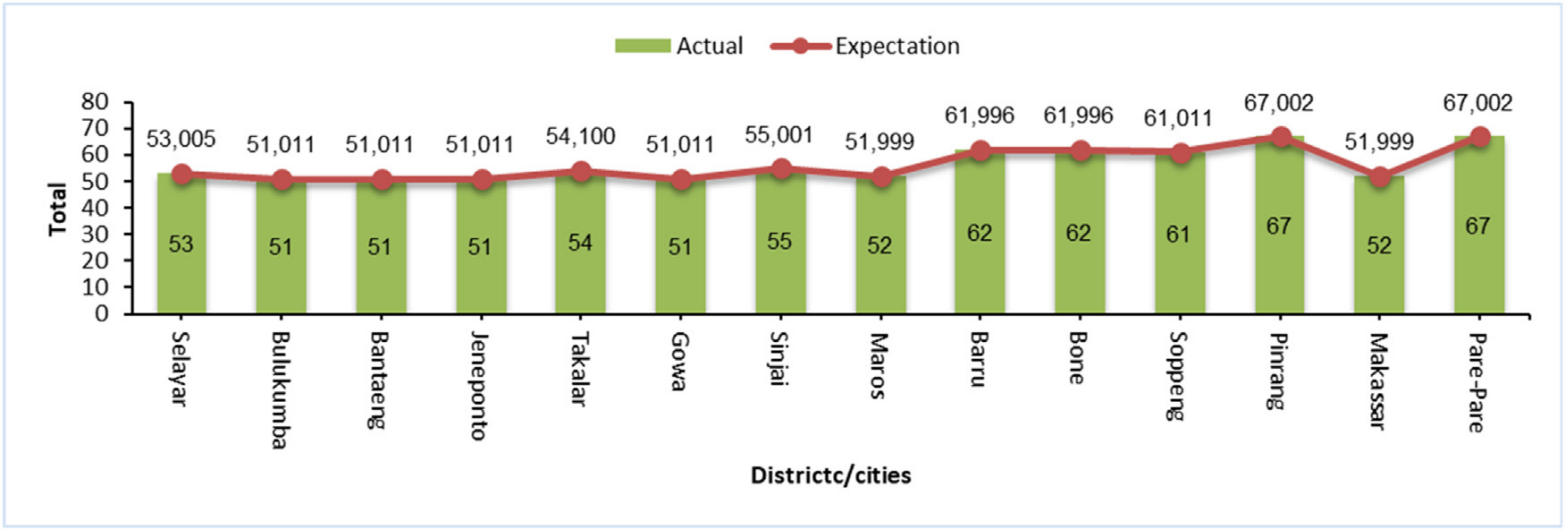
Comparison of the number of actual drought events and the number of suspected droughts using SPI.

**Fig. 6:**
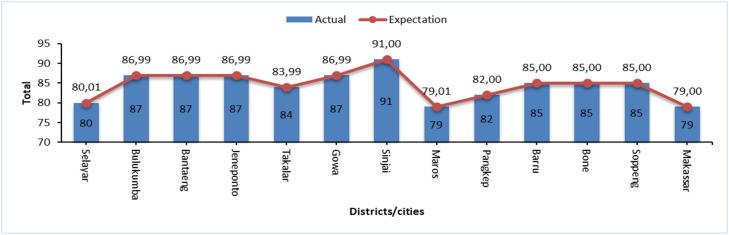
Comparison of the number of actual drought events and the number of suspected droughts using SPEI.

Furthermore, the parameter estimation values are used to estimate the number of drought events that will occur in the future using the Power Law Process expectation function (kumulatif function) in Eq. (27). Before that, the number of drought events that have occurred during the 516 months of observation is estimated. This estimation aims to validate the accuracy of the model and ensure that the estimated number of drought months matches the number of drought events that actually occurred during the period. The results of the comparison between the actual number of events and the predicted number of events in the 516 months of observation in South Sulawesi are shown in Fig. 5, Fig. 6.

### Calculation of the number and timing of meteorological drought events in south sulawesi for the next 12 months

Suppose the time of drought occurrence in each observation area is $t_{i}<t_{2}<\cdots <t_{n}<t$, where t is the end time of the observation period and $N\left(t\right)=\{N,t_{i}<t_{2}<\cdots <t_{n};\left(0,t\right]\}$, N denotes the number of months of drought occurrence in the time interval (0,t]. It is known that the total months observed from 1981 to 2023 are t=516 months. The period of months to be estimated in this study is s=12 months ahead. Thus, the time interval is extended to 516 + 12 = 528.

**Fig. 7:**
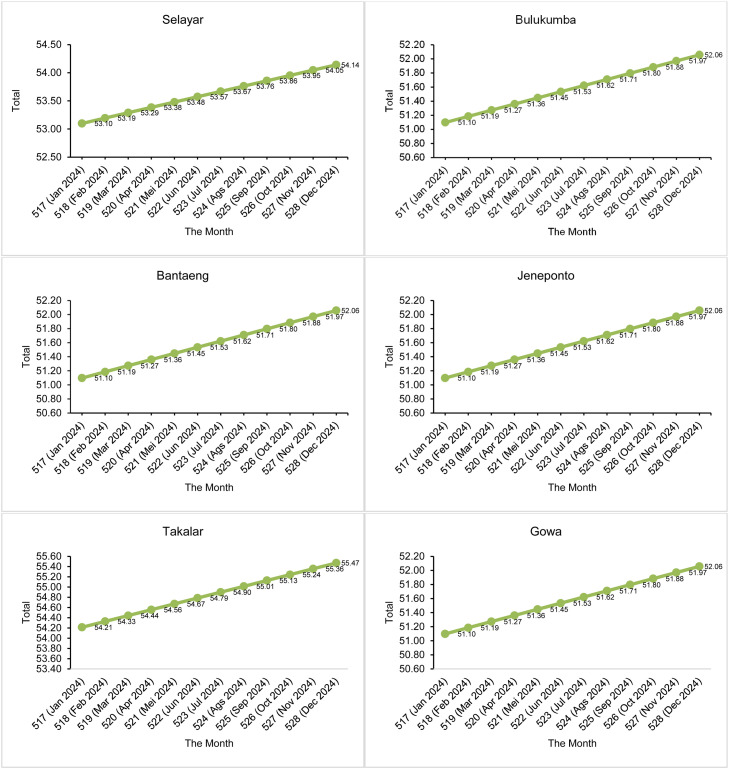

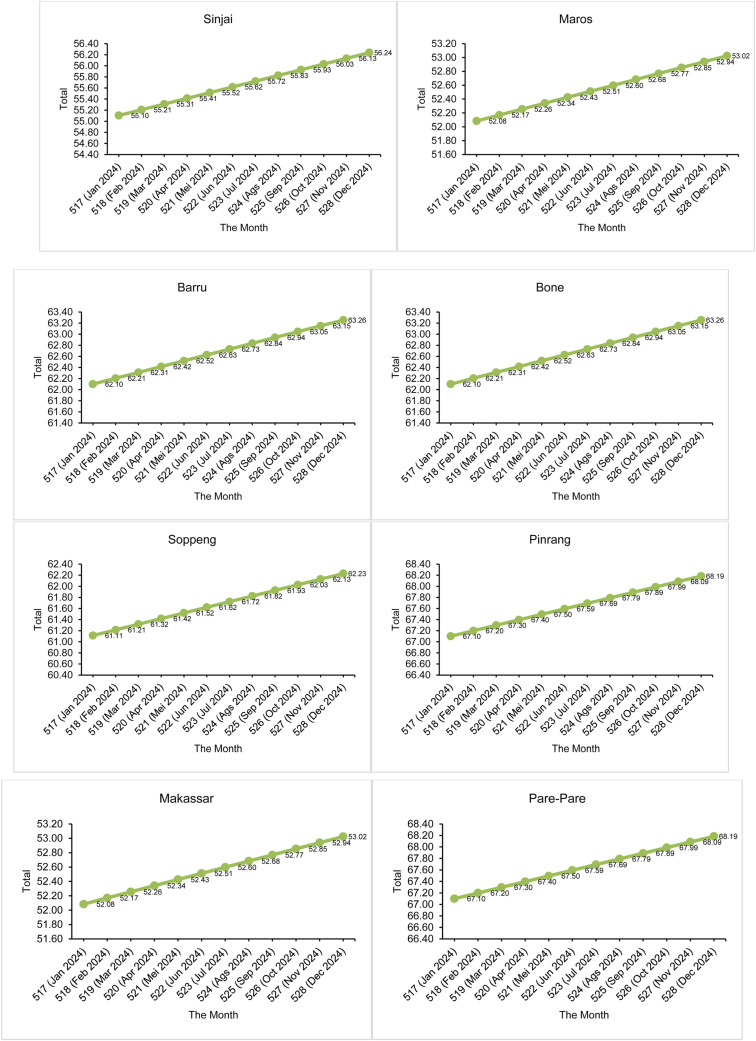
Estimated number of drought events by SPI in the future 12 months.

**Fig. 8:**
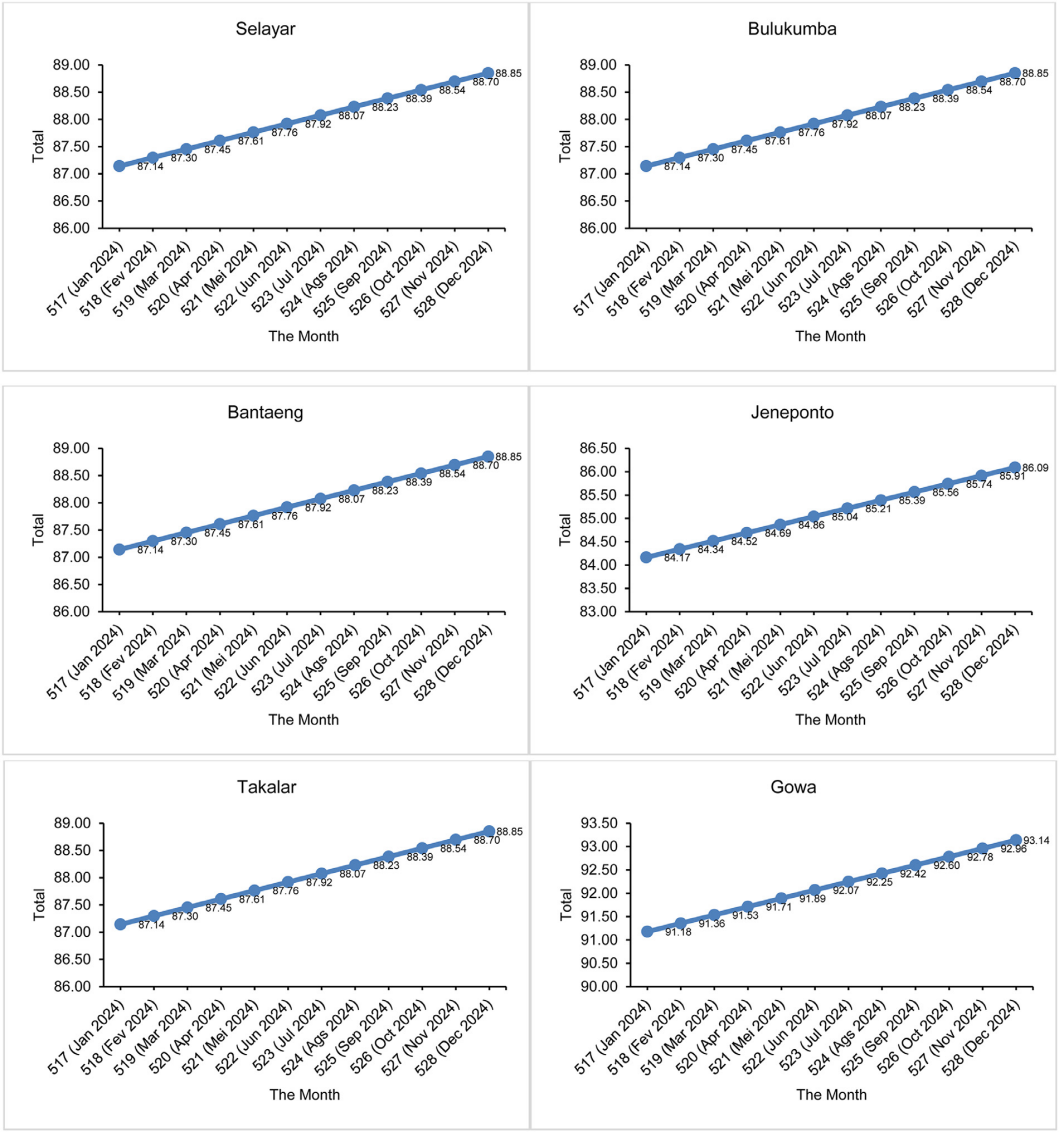

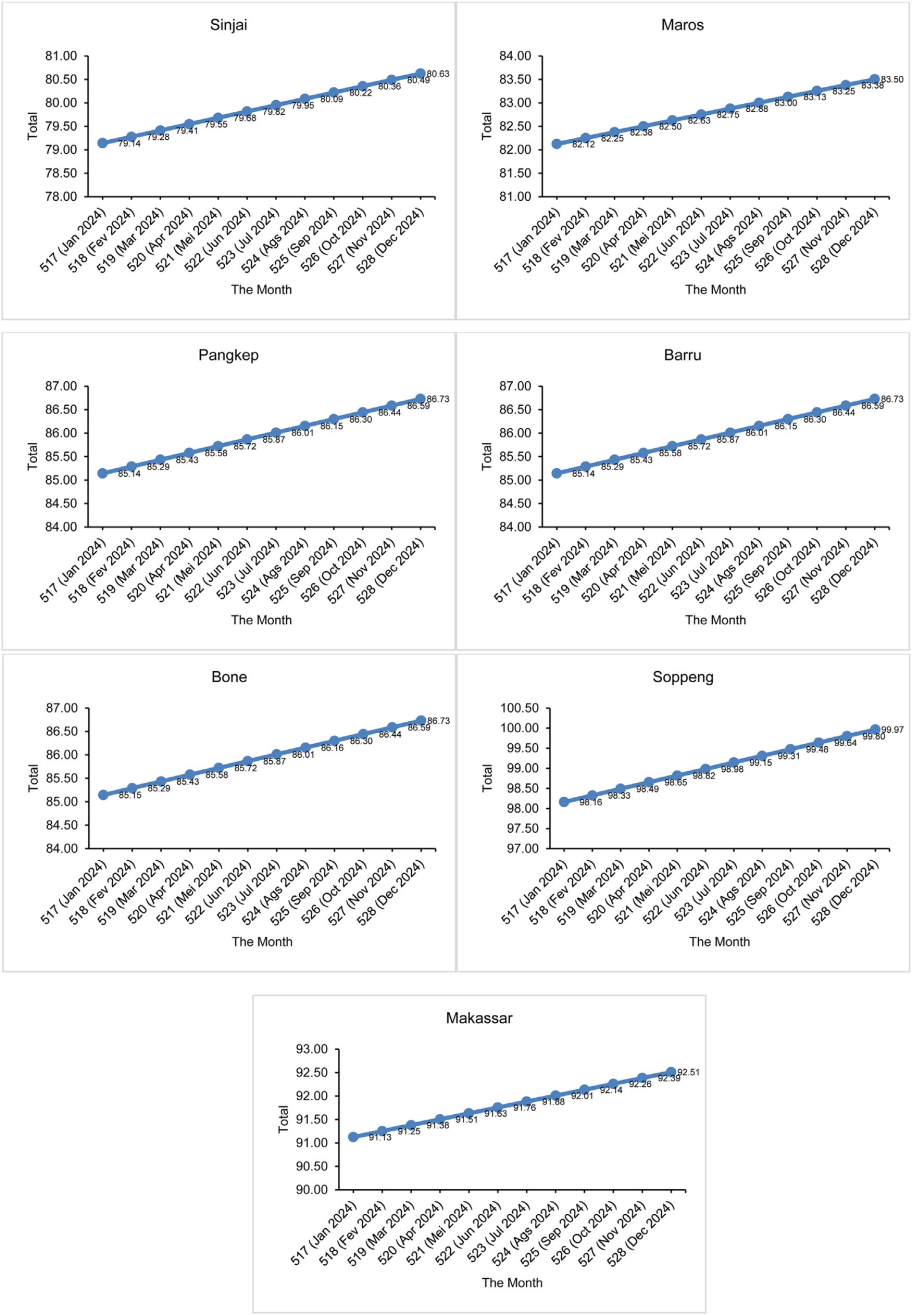
Estimated number of drought events by SPEI in the future 12 months.

In the 516th month, the expected number of drought events has been calculated, which has a good match with the actual number of observations. Next, calculations were made to predict the number of drought events that would occur in the following months, namely month 517 to month 528. The time interval from month 517 to month 528 is the prediction period, where month 517 is January 2024 until month 528 in December 2024. The predicted number of months of SPI drought events in the next 12 months is presented in Fig. 7 and the SPEI in Fig. 8.

If there is an increase in the number of drought events compared to the previous observation period, the increase is considered an indication of the number of drought events that may occur in the future. In other words, the number of drought months predicted in the future is the difference between the predicted number of drought events and the number of drought events that have been observed previously.

Fig. 7 shows that in the next 12 months, all 14 districts/cities in South Sulawesi are expected to experience an increase in the number of drought events based on the 1-month SPI. As a clearer illustration, for example in Selayar District, during the 516-month observation period there were 53 months of drought (see Fig. 5 for the actual number of drought events in Selayar District). After considering an additional 12 months, totaling 528 months, the predicted number of drought events in Selayar District is estimated to increase to 54.14 months (see Fig. 7 for Selayar District). This represents an increase of 1.14 months in the number of drought events compared to the base period (516 months). Overall, Fig. 7 shows that all 14 districts/cities in South Sulawesi are expected to experience an increase in drought occurrence of 1 month compared to the previous observation period. Meanwhile, for Fig. 8, in the SPEI drought index, overall 13 districts/cities in South Sulawesi experienced an increase in the number of drought events by 2 months (we rounded the numbers). Therefore, it can be concluded that the number of droughts that will occur in the next 12 months is 2 months.

**Fig. 9:**
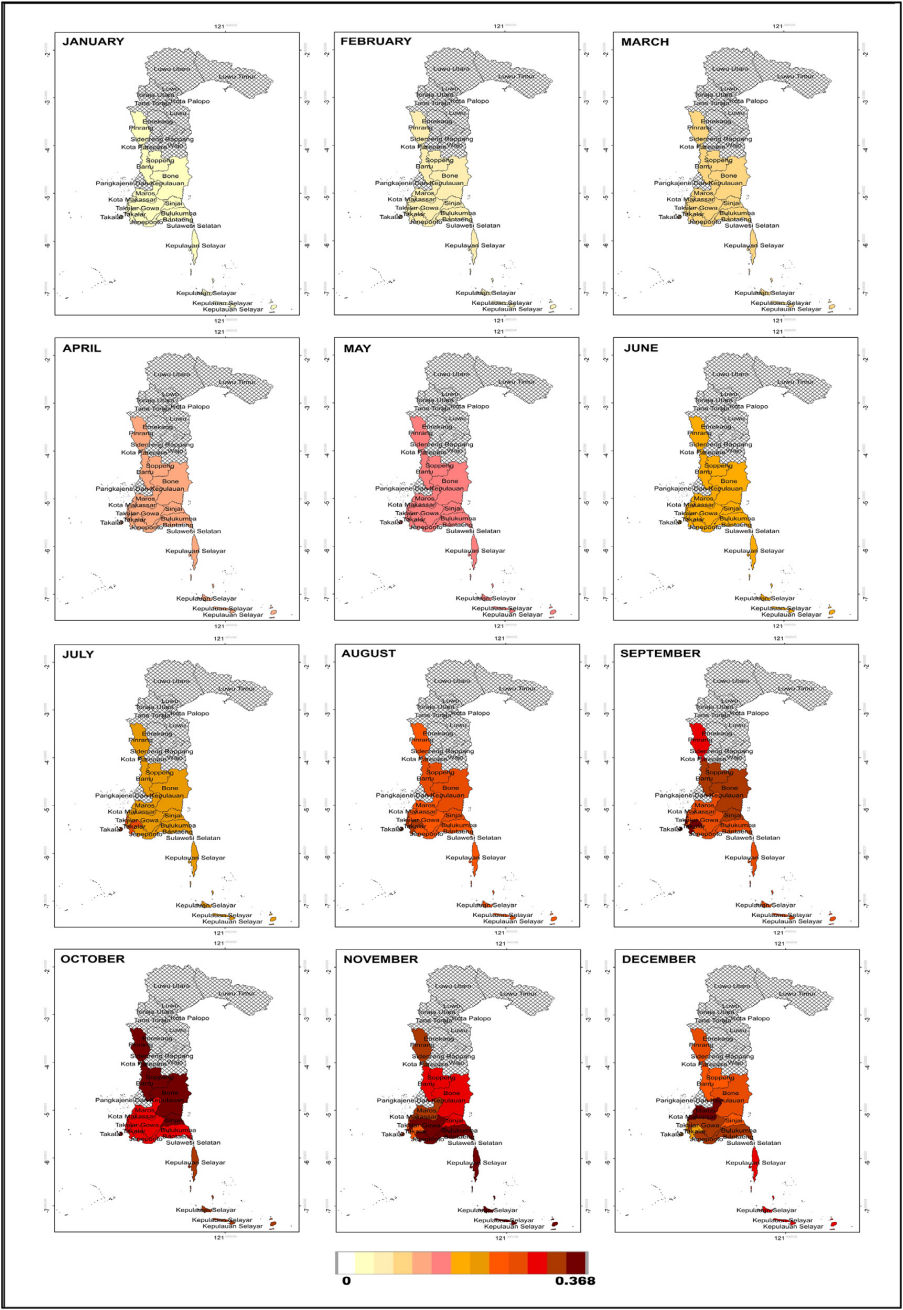
Map of monthly probability of drought event in the next 12 months based on SPI.

**Fig. 10:**
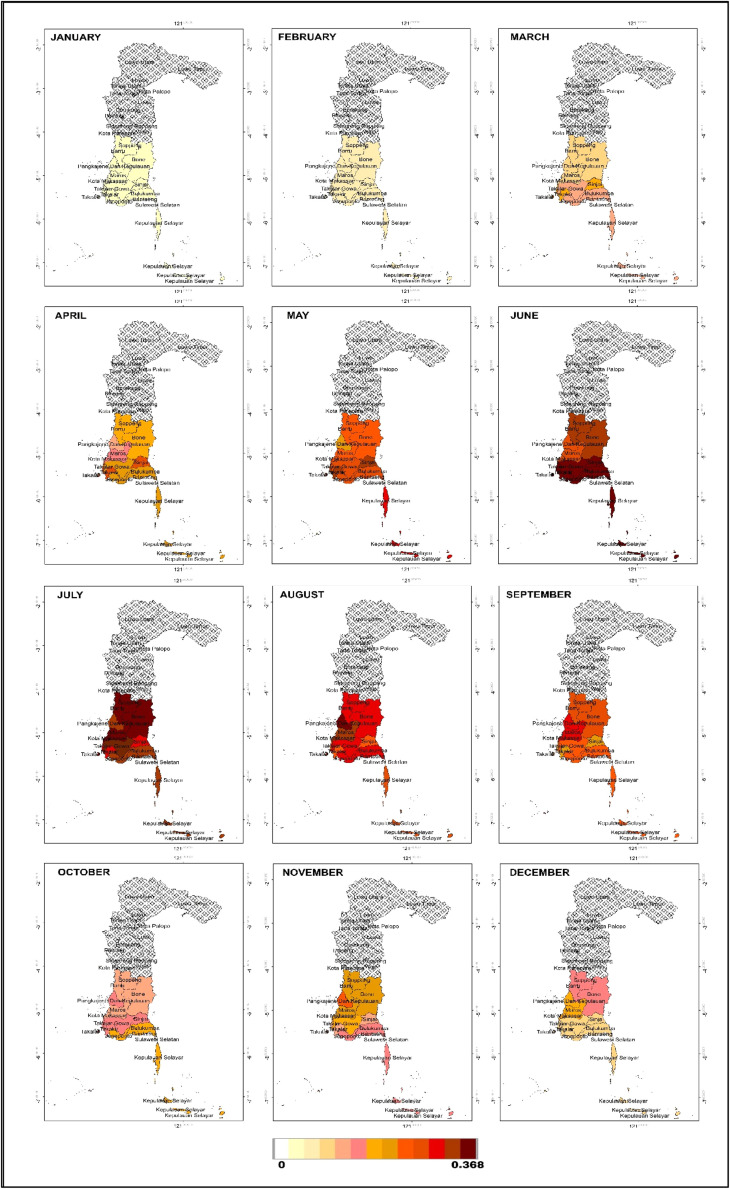
Map of probability of first month drought event in the next 12 months based on SPEI.

**Fig. 11:**
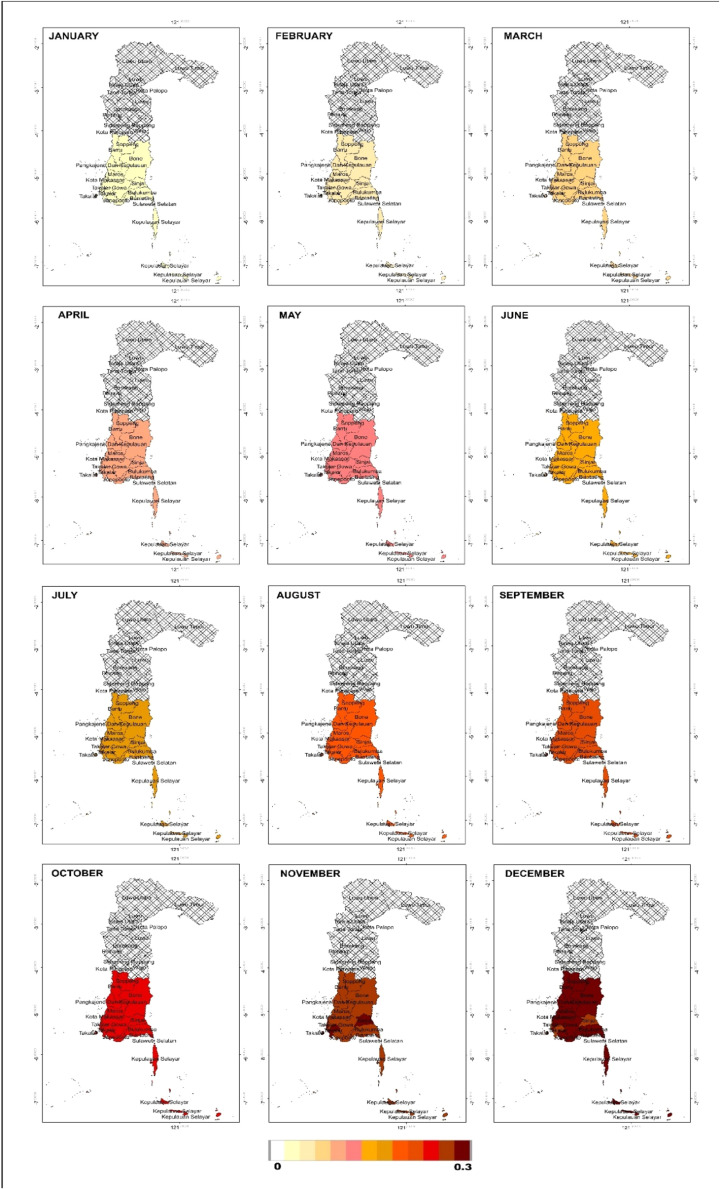
Map of probability of second month drought event in the next 12 months based on SPEI.

As a mitigation and early planning measure, months with potential drought events were mapped based on the probability calculated using the Nonhomogeneous Poisson Process through Eq. (25). In the next 12 months, the number of drought events based on SPI is estimated to be one month, with the highest probability of 0.37. Drought is expected to occur in September in Takalar; October in Barru, Bone, Pare-pare, Pinrang, Sinjai and Soppeng; in November in Selayar, Bulukumba, Bantaeng, Jeneponto and Gowa; and in December in Maros and Makassar. Meanwhile, based on the SPEI, the number of drought events is estimated at two months. In the first month, the highest probability of 0.37 is predicted to occur in June in Selayar, Bulukumba, Bantaeng, Jeneponto, Takalar, Gowa and Sinjai, and in July in Maros, Barru, Bone, Soppeng and Makassar, while for Pangkep it is predicted to occur in August. In the second month, the highest probability of 0.27 is expected in December in 12 regions, except for Sinjai, which experienced drought earlier in November. For more details, the probability values can be seen in Fig. 9 for SPI, and Fig. 10, Fig. 11 for SPEI. Interpretation of the mapping results shows that the months with the highest probability of drought occurrence are marked with darker colours (dark red), indicating areas with higher drought risk.

The implications of these forecast results are very important in water resources management and drought mitigation policies. An increase in the number of drought events, albeit relatively small (1–2 months), could impact water availability for agriculture, households and industry. Therefore, local governments and stakeholders need to consider these predictions in water supply planning, for example by increasing water storage capacity in areas predicted to experience longer droughts, strengthening irrigation systems, and optimising water conservation strategies. Thus, data-driven approaches such as the one used in this study can inform decision-making in managing drought risk more effectively.

## Limitations

None.

## Ethics statements

This article does not involve research with human or animal subjects, and does not include pathology reports or other types of research that require special ethics approval. All data used in this study are publicly available secondary data, obtained from NASA Data Access Power. The authors have ensured that all research procedures were conducted in accordance with applicable ethical standards, and no ethical violations occurred in the collection and use of data.

## CRediT author statement

**Nurtiti Sunusi**: Conceptualization, Methodology, Validation, Formal analysis, Resources, Data curation, Writing-Review & editing, Writing-original draft, Supervision. **Nur Hikmah Auliana**: Methodology, Validation, Visualization, Writing-review & editing.

## Declaration of competing interest

The authors declare that they have no known competing financial interests or personal relationships that could have appeared to influence the work reported in this paper.

## Data Availability

Data will be made available on request.

## Appendix Supplementary materials

Image, application 1
