# Supplementary material for: Assessing SPI and SPEI for drought forecasting through the power law process: A case study in South Sulawesi, Indonesia
Source: MethodsX. 2025 Feb 19;14:103235. doi: 10.1016/j.mex.2025.103235 (PMC11903953; doi:10.1016/j.mex.2025.103235)
Supplement: Supplementary file 1 [file mmc1.docx]

Start

Input: Air Pollution Data

perform forecasting adjustments

Calculating initial forecasting

Create a Markov transition probability matrix

Determine FLR and FLRG

Applications of CLARA to determine medoid and cluster data

Fuzzification

Determining the fuzzy set $A_{i}$

Descriptive analysis of data

Conclusion

Calculating the accuracy of forecasting results

Calculating final forecasting

Finish

Calculating the distance between non-medoids and medoids, adjacent objects will form a cluster.

Calculating the total distance obtained

Determining the number of clusters and selecting samples into data subsets

Application of CLARA to determine medoid and cluster data

Determine the medoid as the number of clusters

Places objects based on the closest distance to the new medoid.

Selecting a new medoid, calculate the distance of the object to the new medoid. If the distance is smaller, then the new medoid replaces the initial medoid.

Iteration?

Return

Calculate the distance of each object 𝑛 to the medoid, close distances will form a cluster.

No

Yes
